# Drain Amylase or Lipase for the Detection of POPF—Adding Evidence to an Ongoing Discussion

**DOI:** 10.3390/jcm9010007

**Published:** 2019-12-19

**Authors:** Benjamin Müssle, Florian Oehme, Stephanie Schade, Marian Sommer, Andreas Bogner, Sebastian Hempel, Julius Pochhammer, Christoph Kahlert, Marius Distler, Jürgen Weitz, Thilo Welsch

**Affiliations:** 1 Department of Visceral, Thoracic and Vascular Surgery, University Hospital Carl Gustav Carus, 01307 TU Dresden, Germany; benjamin.muessle@uniklinikum-dresden.de (B.M.); florian.oehme@uniklinikum-dresden.de (F.O.); stephanie.schade@web.de (S.S.); marian_sommer@yahoo.de (M.S.); andres.bogner@uniklinikum-dresden.de (A.B.); sebastian.hempel@uniklinikum-dresden.de (S.H.); christoph.kahlert@uniklinikum-dresden.de (C.K.); marius.distler@uniklinikum-dresden.de (M.D.); juergen.weitz@uniklinikum-dresden.de (J.W.); 2 Department of General, Visceral, Thoracic, Transplant, and Pediatric Surgery, University Hospital Schleswig-Holstein, 24105 Kiel, Germany; julius.pochhhammer@uksh.de

**Keywords:** pancreatic fistula, drain fluid analysis, amylase, lipase, pancreatic surgery

## Abstract

**Objectives**: A postoperative pancreatic fistula (POPF) is defined as a threefold increase in the amylase concentration in abdominal drains on or after the third postoperative day (POD). However, additional lipase fluid analysis is widely used despite lacking evidence. In this study, drain amylase and lipase levels were compared regarding their value in detecting POPF. **Methods**: We conducted a retrospective study including all patients who underwent pancreatic resections at our center between 2005 and 2016. Drain fluid analysis was performed from day 2 to 5. **Results**: 990 patients were included in the analysis. Overall, 333 (34%) patients developed a POPF. The median amylase and lipase concentrations at POD 3 in cases with POPF were 11.55 µmol/(s·L) (≈13 ×-fold increase) and 39 µmol/(s·L) (≈39 ×-fold increase), respectively. Seven patients with subsequent POPF (2%) were missed with amylase analysis on POD 3, but detected using 3-fold lipase analysis. The false-positive rate of lipase was 51/424 = 12%. A cutoff lipase value at POD 3 of > 4.88 yielded a specificity of 94% and a sensitivity of 89% for development of a POPF. Increased body mass index turned out as risk factor for the development of POPF in a multivariable model. **Conclusions**: Threefold-elevated lipase concentration may be used as an indicator of a POPF. However, the additional detection of POPF using simultaneous lipase analysis is marginal. Therefore, assessment of lipase concentration does not provide added clinical value and only results in extra costs.

## 1. Introduction

Advances in surgical techniques, perioperative management and increasing centralization at high-volume centers have resulted in a significant reduction in perioperative mortality in patients undergoing pancreatic surgery [1,2,3]. However, postoperative morbidity remains challenging and still ranges between 40% and 60%, even at high-volume centers [4]. Postoperative pancreatic fistula (POPF) is the most common septic, harmful and potentially life-threatening complication after pancreatic surgery [5]. The leakage of pancreatic juice and enzymes from the pancreatic anastomosis predisposes for a broad spectrum of associated complications, which may vary from non-clinically relevant to severe systemic events [3,6]. Furthermore, POPF is associated with prolonged hospitalization, increased morbidity and high healthcare costs, and is associated to increased mortality [7]. The incidence of POPF ranges between 5% and 40%, even at high-volume centers [1,8]. A POPF is defined as an amylase concentration in the abdominal drain greater than three times the upper limit of the serum amylase concentration on POD 3 or later, according to the International Study Group on Pancreatic Surgery (ISGPS) [9]. 

However, the definition also has some limitations in that it may fail to detect every clinically relevant POPF (CR-POPF). In some cases, the prediction of POPF is challenging because the amylase concentration of the drainage fluid does not always reliably reflect the real extent of POPF [10,11].

Early detection of CR-POPF may improve clinical management, reduce morbidity and shorten hospitalization, thereby resulting in reduced costs. Therefore, many specialized centers simultaneously analyze the amylase and lipase concentration of drainage fluid for the early detection of POPF. Facy et al. demonstrated that a lipase level higher than 500 units/L yields a sensitivity of 88% for POPF. Moreover, they observed that a lipase level of >1000 units/L predisposes for a worse clinical course and is suggestive of type B or C POPF [12]. Another publication even recommends lipase as the more sensitive biochemical marker for detection of CR-POPF [13]. We therefore compared the use of postoperative drainage fluid amylase and lipase concentration and their prognostic impact on the diagnosis of POPF in a large retrospective cohort. The primary endpoint was to assess false negative POPFs according to the amylase assessment, which were detectable by lipase drain analysis. Furthermore, we sought to determine if there is a threshold at which the drain fluid analysis of lipase is superior for prediction of POPF compared with amylase levels. In the present study, we used the well-established cutoff value of amylase for the definition of POPF during the consecutive clinical course, although there are limitations in investigating a lipase-based test against this standard. In addition, we investigated predisposing factors for POPF using the underlying cohort.

## 2. Methods

### 2.1. Study Cohort

All patients who underwent pancreatic resections for malignant and benign diseases between February 2005 and October 2016 at the Department of Visceral, Thoracic and Vascular Surgery, University Hospital Carl Gustav Carus, TU Dresden were included in this retrospective analysis. The study was approved by the local ethics committee (decision no. 294072017). The pancreatic surgery comprised the following surgical procedures: pylorus-preserving pancreatoduodenectomy (PPPD), duodenum-preserving pancreatic head resection (DPPHR), classic partial duodenopancreatectomy (cPD), segmental pancreatectomy, distal pancreatectomy (DP), enucleation and ampullectomy.

All clinical, biochemical and radiological data were retrospectively obtained from a prospective database. The collected data included patient demographics (age, sex), the American Society of Anesthesiologists Score (ASA), weight, height, body mass index (BMI), indication for surgery, preoperative treatment, type of operation, postoperative clinical data, histopathological data, radiological data, the need for additional percutaneous drainage, levels of the pancreatic enzymes in the drainage fluid and the corresponding serum values, postoperative complications, morbidity and mortality.

### 2.2. Pancreatic Enzyme Analysis

POPFs were defined according to the latest ISGPS definition [9]. Based on the updated definition, a POPF is defined as a drain output of any measurable volume of fluid with an amylase level >3 times the upper limit of institutional normal serum amylase activity, and is classified in three different grades. In absence of clinical symptoms or consequences, this condition is defined as a biochemical leak (BL). In contrast, grades B and C necessitate a change in the postoperative management and are clinically relevant (CR) fistulas. Grade B requires a prolonged abdominal wound drainage > 3 weeks or an interventional/endoscopic drainage. Grade C refers to a severe clinical condition, which requires a reoperation or leads to organ failure and/or mortality triggered by the POPF. [9] Amylase and lipase levels were routinely measured in the abdominal drainage fluid and serum between POD 2 and 5. The measurement was repeated in patients where the presence of POPF was suspected, e.g., due to changes in drainage fluid quality or the interventional drainage of a perianastomotic fluid collection. The enzyme assay was performed using the Roche Cobas 8000 Analyzer (Roche, Mannheim, Germany). Threefold-elevated amylase levels > 2.64 µmol/(s·L) (standard value < 0.88 µmol/(s·L)) and lipase levels > 3.0 µmol/(s·L) (standard value < 1 µmol/(s·L)) in the drainage fluid were considered to be increased.

### 2.3. Statistical Analysis

The statistical data analysis was performed using the IBM SPSS Statistics program (version 23, SPSS Inc., Chicago, IL, USA) and the graphical representation with the GraphPad Prism software package (version 6.01, GraphPad Software, Inc., La Jolla, CA, USA). Sensitivity and specificity for the potential diagnostic performance of the drain fluid concentration of lipase from POD 2 to 5 were assessed using receiver operating characteristic (ROC) curves. The optimal cutoff point, maximizing the balance between sensitivity and specificity, was determined by calculation of the Youden index. This can be defined as Y = Sensitivity + Specificity – 1 and ranges from 0 to 1 for each cutoff point. Assuming an equal importance of sensitivity and specificity, a cutoff point is identified based on the highest Youden index [14]. The χ^2^ test was used for comparison of categorical variables in univariate analysis. Univariate analysis was performed for the various preoperative risk factors. Statistically significant variables demonstrated in the univariate analysis were incorporated into a stepwise backwards multivariate logistic regression analysis, to identify independent factors for POPF after pancreatic resections. The values were presented as the median and interquartile range (IQR), unless otherwise indicated. A *p*-Value less than 0.05 was considered statistically significant. Univariate analyses were applied for multiple testing, controlling the false discovery rate using the Benjamini–Hochberg procedure [15].

## 3. Results

### 3.1. Patient Demographics

Nine hundred and ninety (990) patients (411 females and 579 males) with a median age of 64 years (IQR 53–73) who underwent pancreatic surgery were included in the analysis (Table 1). The resections were combined with a simultaneous portal vein resection in 171 cases (17.1%). Three hundred and thirty-two patients received a multi-visceral resection including 68 simultaneous resections of the spleen during DP. A pancreatic ductal adenocarcinoma was the most common reason for pancreatic resection (35.6% of all cases) (Table 2). A PPPD was the most frequent operation (Table 3).

### 3.2. Drain Fluid Analysis for the Diagnosis of Postoperative Pancreatic Fistula

**Figure 1:**
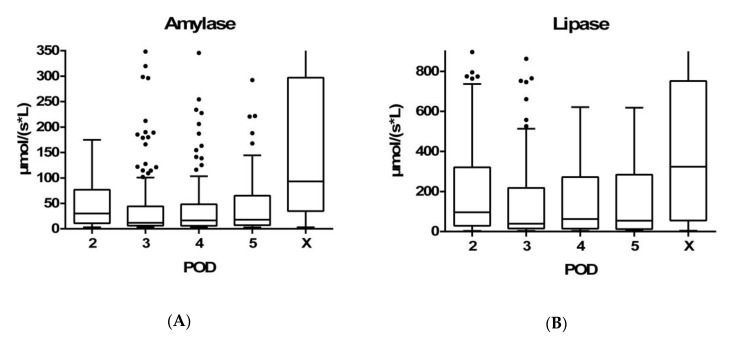
Box plot demonstrating amylase and lipase concentration in the drain fluid in patients with POPF. (**A**) A threefold-elevated amylase concentration was found in 251/242/135/99/39 patients on PODs 2–5, and on the day of POPF diagnosis (X). The amylase concentrations in the drainage fluid were determined in 786/669/450/404/52 patients from POD 2 to POD X. (**B**) A threefold-elevated lipase concentration was found in 246/237/146/123/49 patients on PODs 2–5 and on the day of POPF diagnosis (X). The lipase concentrations in the drainage fluid were determined in 776/661/448/401/63 patients from POD 2 to POD X. The Tukey method was used for plotting the whiskers and outliers. Amylase and lipase concentrations were considered normal <0.88 µmol/(s·L) and <1 µmol/(s·L), respectively. POPF = postoperative pancreatic fistula, POD = postoperative day, X = day of interventional insertion of a drain or reoperation. X-axis: POD 2-X; Y-axis: amylase concentration in the drainage fluid (µmol/(s·L)).

**Figure 2:**
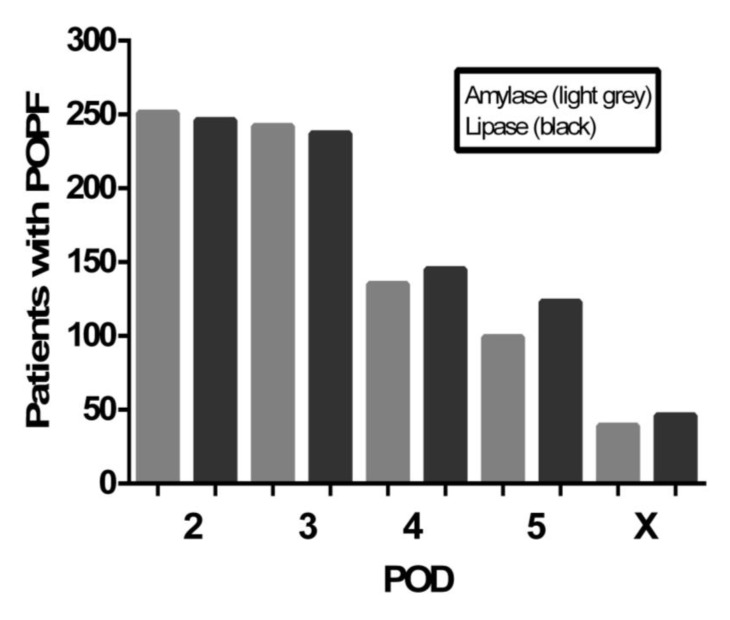
Number of patients with POPF and threefold-elevated amylase and lipase concentration in the drainage fluid on PODs 2–5 and on the day of POPF diagnosis. POPF = postoperative pancreatic fistula, POD = postoperative day, X = day of interventional insertion of a drain or reoperation. X-axis: POD 2-X; Y-axis: number of patients with threefold-elevated amylase/lipase.

The amylase concentrations in the drainage fluid were determined in 786/669/450/404 of patients from POD 2 to 5, respectively, and in 52 patients on the day of POPF diagnosis (X) (Figure 1A). A threefold-elevated amylase concentration was found in 251/242/135/99 (i.e., 32%/36%/30%/25%) of the patients on PODs 2–5, respectively, and in 39 patients on POD X (Figure 2). In comparison, the lipase concentrations in the drainage fluid were determined in 776/661/448/401 of the patients on PODs 2–5, respectively, and in 63 patients on POD X (Figure 1B). A threefold-elevated lipase concentration was found in 246/237/146/123 (i.e., 32%/36%/33%/31%) of the patients on PODs 2–5, respectively, and in 49 patients on the POD X (Figure 2). 

Overall, 333 (34%) patients developed a POPF according to the ISGPF definition. Of these, 229 (23%) patients presented with a BL, 63 (6%) patients developed a grade B POPF, and 41 (4%) a grade C POPF. The median drain amylase value of patients with a POPF at POD 3 was 11.55 (IQR: 6.13–43.04) (≈13 ×-fold increase). In contrast, the median drain amylase of patients without a POPF was 0.29 (0.09–0.79). The median lipase concentration at POD 3 was 39.29 (IQR: 15.17–217.42) (≈39 ×-fold increase) in patients with POPF and 0.34 (0.12–0.33) in patients without clinical evidence of a POPF.

The highest amylase and lipase concentration in the drainage fluid was found either on POD 2 or on the day the interventional drainage was placed (amylase 27.37 (9.76–74.89) and 62.48 (9.02–282.87), respectively; lipase 89.14 (25.45) and 214.83 (31.93–618.45), respectively) (Figure 1A,B). 

### 3.3. Test for POPF on POD 3

**Figure 3:**
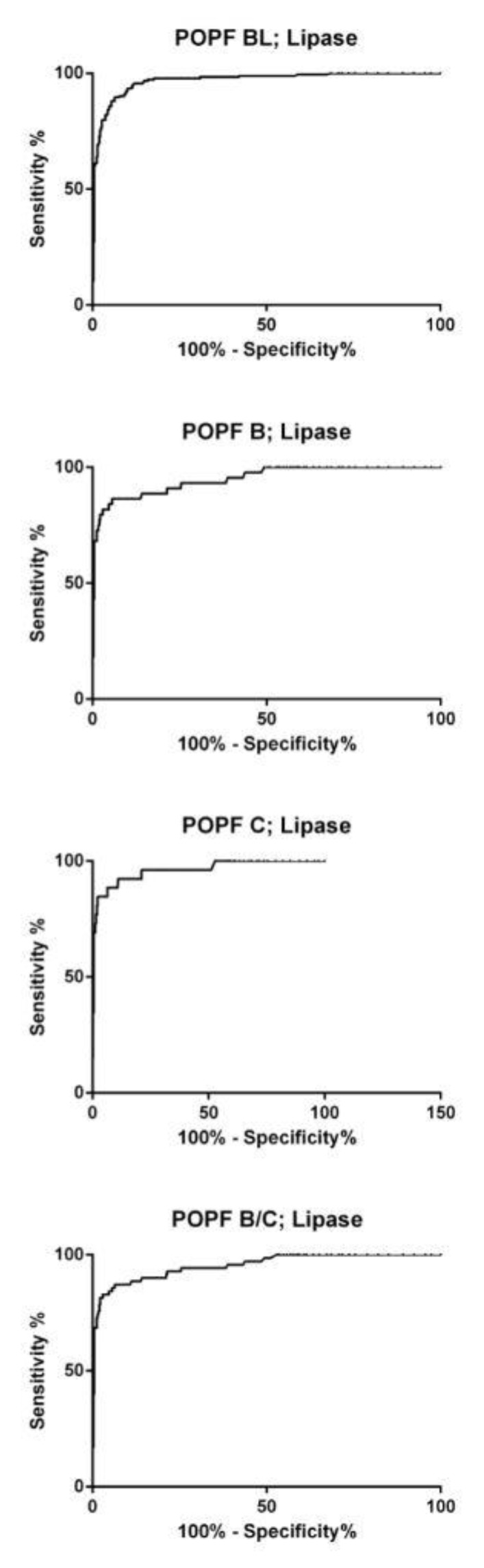
Receiver operator characteristic (ROC) curve for cutoff analysis of drain fluid lipase at POD 3 according to the different ISGPS POPF grades. POPF from BL to grade C and the combination of B and C (CR-POPF) is shown for lipase. ROC = receiver operator characteristic, ISGPS = International Study Group in Pancreatic Surgery, POPF = postoperative pancreatic fistula, BL = biochemical leakage, CR-POPF = clinical relevant-postoperative pancreatic fistula.

We calculated the predictive test statistics for the amylase test on POD 3 for prediction of a POPF during the entire postoperative course. On POD 3, amylase was negative, i.e., not threefold-elevated, in two patients with a subsequent BL, in three patients who later developed a grade B POPF (diagnosed on PODs 5, 7, 12, respectively), and in two patients who developed a grade C POPF (diagnosed on POD 8 and 10, respectively). The false-negative rate of the amylase test on POD 3 was 7/249 = 2.8% (sensitivity 235/242 = 97.1%). Specificity of amylase test was necessarily 100%, since definition of ISGPS was applied. However, analysis of the drainage fluid revealed a threefold-elevated lipase concentration in all these seven patients on POD 3. In comparison, only three patients with a BL according to the amylase definition (ISGPS) had no threefold-elevated lipase levels in the drain fluid (false-negative rate 3/240 = 1.3%, sensitivity 234/237 = 98.7%). Interestingly, 51 patients without a POPF had a threefold-increased lipase concentration in the drainage fluid at POD 3 (false positive rate = 51/424 = 12.0%; specificity 89.2%). The positive predictive value (PPV) for lipase on POD3 for the detection of POPF was 82.2%. During the early postoperative course, almost all patients with increased amylase concentrations in the drainage fluid also had increased lipase concentrations (Figure 3).

ROC curve analysis for lipase at POD 3 showed a cutoff value > 4.88 (≈5 ×-fold increase) and yielded a sensitivity of 89% and a specificity of 94% (area under the curve (AUC) = 0.96; confidence interval (CI) = 0.95–0.97; *p* = <0.0001) for all patients independent of the POPF grade.

Subgroup analysis revealed a sensitivity of 92% and a specificity of 88% for lipase (cutoff > 3.08, AUC = 96; CI = 0.95–0.98; *p* = <0.0001) for patients with a BL. For patients with a grade B fistula, the sensitivity and specificity of lipase were 89% and 86%, respectively (cutoff 2.84, AUC = 0.94; CI = 0.91–0.98; *p* = <0.0001). In patients with a grade C fistula, the sensitivity and specificity of lipase were 88% and 94%, respectively (cutoff > 4.88, AUC = 0.96; CI = 91–1.00; *p* = <0.0001). Optimal lipase cutoff in patients with a CR-POPF (grade B/C) was > 4.88 with a sensitivity of 87% and specificity of 94% (AUC = 0.95; CI = 0.92–0.98; *p* < 0.0001) (Figure 3). 

During the study period, we performed 2349 lipase drain fluid tests between POD 2 and 5. At a current cost of EUR 0.95/per lipase test, the total costs of routine lipase analysis came to EUR 2231.55.

### 3.4. Morbidity and Mortality

Patients with a POPF had a significantly longer hospital stay (23 days (16–34.2) vs. 17 (13–23) *p* = <0.05). There was a significant increase in postoperative complications such as bile duct leakage, surgical site infection (SSI), intraabdominal abscess formation, pancreatitis, delayed gastric emptying (DGE), pneumonia, pleural effusion, post-pancreatectomy hemorrhage (PPH) and intraluminal bleeding (Table 4). Furthermore, the 30-day mortality rate was significantly higher in the POPF group (4.5% vs. 2.8%; *p* = 0.02). The 30-day mortality rate was 4.5% (*n* = 15 of 333 patients). Eleven of these patients died due to POPF-associated septic complications and PPH. In addition, another patient passed away due to postoperative liver failure in liver cirrhosis, and three patients died as a result of cardiac arrest and consequent multi-organ failure.

### 3.5. Risk Factors for POPF

A univariate comparison of variables showed that BMI, distal bile duct and ampullary cancer, IPMN and distal pancreatectomies were associated with a significantly increased risk of POPF. In contrast, diabetes, tobacco and alcohol abuse, PPPD, DPPHR, portal vein resection, pancreatitis, and pancreatic ductal adenocarcinoma were associated with a decreased risk of developing a POPF.

In a multivariate analysis, only BMI remained a significant risk factor for POPF (odds ratio (OR) 1.084; 95% CI 1.047–1.123; *p* = <0.05). There was a negative trend for male gender and POPF (OR 0.729; 95% CI 0.526–1.011; *p* = 0.058). Protective factors with a decreased incidence of POPF were diabetes (OR 0.644; 95% CI 0.456–0.909; *p* = <0.05), alcohol (OR 0.730; 95% CI 0.478–1.117; *p* = <0.05), PPPD (OR 0.616; 95% CI 0.452–0.839; *p* = <0.05), portal vein resection (OR 0.591; 95% CI 0.367–0.950; *p* = <0.05), pancreatitis (OR 0.422; 95% CI 0.260–0.687; *p* = <0.05) and PDAC (OR 0.356; 95% CI 0.247–0.514; *p* = <0.05).

## 4. Discussion

The occurrence of a POPF is the primary cause of sepsis and other severe complications after pancreatic surgery [16]. A POPF can cause a broad spectrum of complications, ranging from non-clinically relevant to severe systemic and life-threatening complications [17,18]. According to the revised guidelines of the ISGPS, a POPF is defined as a threefold increase in amylase in the drain fluid when compared to the normal serum value measured at postoperative day three or later [9]. 

The vast majority of high-volume pancreatic centers routinely investigate drain fluid amylase and lipase concentration to detect POPF. However, it is still unclear whether drain fluid lipase is superior to amylase in detecting POPF, or if it provides an additional diagnostic benefit for patients after pancreatic surgery. Furthermore, as a biochemical marker, serum lipase has been reported to have superior sensitivity and specificity—and greater overall accuracy—than amylase in predicting acute pancreatitis [19]. We therefore conducted this retrospective study, to address this unsolved problem and provide further data about the usefulness of simultaneous lipase and amylase measurement for the early detection of POPF. Furthermore, we wanted to identify predisposing factors for the development of POPF.

In the present study, the overall incidence of POPF reached 34%, which is in good accordance with data from numerous other studies. However, we found a relatively high incidence of BL (23%), whereas the incidence of CR-POPF was low in comparison to other studies [16]. The retrospective data analysis of the present study may explain this discrepancy.

In the case of DP, we observed an increased lipase or amylase concentration in 48% of all cases. However, in the vast majority (35% of all cases), this increased concentration was associated only with a BL.

One of our major findings shows that most patients with POPF had threefold-elevated amylase and lipase concentrations in the drainage fluid between PODs 2 and 5.

The highest concentration was measured on POD 2, or on the day when an interventional drain was placed. It is known that patients who undergo pancreatic surgery have increased enzyme levels in the drainage fluid in the first days after an operation. This might be caused by spilled pancreatic enzymes during the operation, or by transient leakage from the stitch holes of the pancreatic anastomosis or the transection surface.

Single studies have reported contradictory results regarding the correlation between lipase concentration in the drainage fluid and the prediction of POPF.

A previous study claims that lipase has a higher sensitivity and specificity than amylase for detecting a CR-POPF (grade B or C). The authors concluded that the analysis of lipase results in a higher detection rate of CR-POPF than the measurement of amylase [12]. In this study, thresholds were established that favored sensitivity because the focus of the author’s investigation was to detect patients at high risk of developing POPF. 

Another retrospective study analyzed 68 patients. A total of 11 patients developed a CR-POPF. Amylase had a sensitivity of 81.8% and a specificity of 69.2%, while lipase had a sensitivity of 91% and a specificity of 64.9%. The authors proposed that drain fluid analysis is not sufficiently sensitive and specific to exclude CR-POPF. Nevertheless, they assumed that lipase might be more sensitive as a biochemical marker for routine detection of CR-POPF [13]. 

However, the results of these two studies must be interpreted critically due to the small number of cases.

In our series, we calculated an optimal cutoff value for lipase analysis according to the Youden index, which is minimally higher than the classic threefold increase. Although three patients with a grade B POPF and two patients with a grade C POPF with a threefold-elevated lipase were amylase negative at POD 3, an earlier detection of the POPF would not have altered the clinical management of the respective patients. 

At our institution, the cost per sample for the drainage fluid analysis was the same for amylase (EUR 0.95) and lipase (EUR 0.95). Eliminating the routine determination of lipase would result in a small reduction in annual costs.

The present study demonstrated that hospital stays were significantly longer—and postoperative complications and mortality significantly higher—in patients with POPF.

Many studies showed a relevant association between different factors (gender, age, nutritional status, BMI, underlying pathology, texture of the pancreatic remnant, diameter of the pancreatic duct, time of operation, experience of the surgeon, blood loss, type of anastomosis and closure of the pancreatic stump) and POPF [20,21,22].

In addition, we found a significant association between the histopathological diagnosis and POPF. These conditions included distal bile duct cancer, ampullary cancer and IPMN. The listed pathologies are often associated with a soft pancreatic texture, which increases the risk of POPF. On the other hand, PDAC or chronic pancreatitis supports the presence of a hard pancreatic texture. This results in a relatively decreased incidence of POPF. Furthermore, alcohol and tobacco abuse are protective factors which result in a decreased incidence of POPF. Both risk factors can induce chronic pancreatitis, resulting in a hard pancreatic texture. Our data support this fact because alcohol and tobacco consumption was more common in the group of patients with chronic pancreatitis.

Diabetes has been shown to be a risk factor for postoperative complications in various surgical disciplines, including gastrointestinal surgery [23,24,25].

Interestingly, our study shows that diabetes does not support the development of POPF, which is in line with other studies. Diabetes results in a transformation of the pancreatic gland. People with diabetes have a significantly less fatty, more fibrotic and thus firmer pancreatic gland texture [26,27]. As previously described, we observed that an increased BMI serves as a predisposing factor for POPF [21]. 

### 4.1. Limitations of the Study

A limitation of the retrospective study design is the lack of data of the amylase and lipase concentration in the drainage fluid of all patients on each POD. Furthermore, we used the established amylase-based ISGPS fistula definition as the “gold standard” for comparison with lipase analysis. This may bias our results, as we could not independently analyze amylase or lipase measurements. This approach would require an additional objective factor as a “gold standard” that would allow us to evaluate these two markers (amylase and lipase) independently.

### 4.2. Conclusion

In conclusion, the current study underscores that routine drain fluid analysis of lipase for the discrimination of POPF in pancreatic surgery patients is not necessary and provides no relevant benefit for diagnosis or clinical management. Indeed, our results support that a threefold-increased lipase concentration has a comparable sensitivity and specificity than amylase in detecting POPF. In more detail, 51 patients without POPF had a threefold-increased lipase in the drainage fluid (false-positive rate 51/424 = 12%). On the other hand, lipase analysis causes extra costs. Routine determination of lipase concentration in the drainage fluid does not seem to provide a clinically relevant advantage for patient management in clinical practice. In line with other studies, our data confirm that BMI and soft pancreatic texture predispose for POPF, whereas diabetes and hard pancreatic gland texture do not increase the risk of a POPF.

## Figures and Tables

**Table 1:** Characteristics of patients who underwent pancreatic surgery between February 2005 and October 2016.

	Total *N* = 990	Median°	IQR	No POPF *N* = 657	POPF *N* = 333	Univariate Analysis	Multivariate Analysis		
	(*n* (%)) *			(*n* (%)) *	(*n* (%)) *	*p*-Value	OR	95% CI	*p*-Value
Age	-	64	53–73	64°	65°	0.804			
ASA-Score	-	2	2–3	2°	2°	0.757			
BMI (kg/m²)	-	25	22–28	24°	26°	<0.001	1.084	1.047–1.123	<0.001
Sex		-	-	273 (41.5)	138 (41.4)	0.973			
Female	411 (41.5)				195 (58.5)				
Male	579 (58.5)			384 (58.4)			0.729	0.526–1.011	0.058
Comorbidities	682 (68.9)	-	-	450 (68.4)	232 (69.6)	0.706			
Renal insufficiency	97 (9.8)			58 (8.8)	39 (11.7)	0.149			
Diabetes mellitus	294 (29.7)			213 (32.4)	81 (24.3)	0.008			
Coronary diseases	93 (9.4)			62 (9.4)	31 (9.3)	0.948			
Hypertension	549 (55.5)			352 (53.5)	197 (59.1)	0.095	0.644	0.456–0.909	0.012

OR = odds ratio; CI = confidence interval; ASA = American Society of Anesthesiologists Score; BMI = body mass index. * Data are presented as *n* (%), if not indicated otherwise. ° Data are presented as median.

**Table 2:** Prevalence of postoperative pancreatic fistula (POPF) according to corresponding histology.

Entity	Total	No POPF	POPF	Univariate Analysis	Multivariate Analysis		
	(*n* (%))	(*n* (%))	(*n* (%))	*p*-Value	OR	95% CI	*p*-Value
Malign	638 (64.4)	426 (64.8)	212 (63.6)	0.737			
Benign	340 (34.4)	224 (34.0)	116 (34.8)	0.804			
PDAC	352 (35.6)	278 (42.3)	67 (20.1)	<0.001	0.356	0.247–0.514	<0.001
IPMN or cystic neoplasm	118 (11.9)	63 (9.5)	55 (16.5)	0.002			
Distal bile duct cancer	156 (15.8)	79 (12.0)	77 (23.1)	0.043			
Duodenal cancer	12 (1.2)	6 (0.9)	6 (1.8)	0.231			
Pancreatitis	196 (19.8)	152 (23.1)	44 (13.2)	<0.001	0.422	0.260–0.687	0.001
Others	156 (15.7)	79 (12.0)	84 (25.2)				

OR = odds ratio; CI = confidence interval; PDAC = pancreatic ductal adenocarcinoma; IPMN = intraductal papillary mucinous neoplasm. * Data are presented as *n* (%), if not indicated otherwise.

**Table 3:** Prevalence of postoperative pancreatic fistula (POPF) according to type of operation.

Surgery	Quantity	No POPF	POPF	Univariate Analysis	Multivariate Analysis		
	(*n* (%)) *	(*n* (%)) *	(*n* (%)) *	*p*-Value	OR	95% CI	*p*-Value
PPPD	578 (58.3)	419 (72.5)	159 (27.5)	<0.001	0.616	0.452–0.839	0.002
DPPHR	59 (6.0)	47 (79.7)	12 (20.3)	0.026			
cPD	105 (10.6)	64 (61.0)	41 (39.0)	0.215			
PSR	17 (1.7)	4 (23.5)	13 (76.5)	<0.001			
DP	185 (18.7)	96 (51.9)	89 (48.1)	<0.001			
Enucleation	21 (2.1)	9 (42.9)	12 (57.1)	0.021			
Ampullectomy	25 (2.6)	17 (68)	8 (32)	0.754			
Multivisceral resection	332 (33.5)	216 (65.1)	116 (34.9)	0.527			
Portal vein resection	171 (17.3)	139 (81.3)	32 (18.7)	<0.001	0.591	0.367–0.950	0.030

OR = odds ratio; CI = confidence interval; PPPD = pylorus preserving duodenpancreatectomy; DPPHR = duodenum-preserving pancreatic head resection, cPD = classic partial duodenpancreatectomy, PSR = pancreatic segmental resection, DP = distal pancreatectomy * Data are presented as *n* (%), if not indicated otherwise. The resections were combined with a simultaneous multi-visceral resection in 33.5% and with a portal vein resection in 17.3% of cases.

**Table 4:** Morbidity in the subgroups of pancreatic resection with and without POPF§.

Complications	No POPF	POPF	Overall	Univariate Analysis
	(*n* (%)) *	(*n* (%)) *	(*n* (%)) *	*p*-Value
Patients	657 (67.4)	333 (33.6)	990 (100)	
Intrabdominal abscess	46 (7.0)	72 (21.6)	117 (11.8)	<0.001
Cholangitis	7 (1.1)	6 (1.8)	13 (1.3)	0.121
Surgical site infection	113 (17.2)	85 (25.5)	198 (20.0)	0.002
Bile duct leakage	50 8 (7.6)	91 (27.3)	141 (14.2)	<0.001
PPH	36 (5.5)	60 (18.0)	96 (9.7)	<0.001
A	1 (2.8)	1 (1.7)	2 (2.1)	
B	23 (63.9)	31 (51.7)	54 (56.3)	
C	11 (30.6)	26 (43.3)	37 (38.5)	
Gastrointestinal hemorrhage	21 (3.2)	31 (9.3)	52 (5.3)	<0.001
DGE	159 (24.2)	119 (35.7)	278 (28.1)	<0.001
A	91 (57.2)	1 (1.7)	152 (54.7)	
B	50 (31.4)	31 (51.7)	78 (28.1)	
C	18 (11.3)	26 (43.3)	48 (17.3)	
Pneumonia	22 (3.3)	40 (12.0)	62 (6.3)	<0.001
Pleural effusion	170 (25.9)	162 (48.6)	332 (33.5)	<0.001
Pulmonary embolism	16 (2.4)	9 (2.7)	25 (2.5)	0.783
Deep venous thrombosis	24 (3.6)	12 (3.6)	36 (3.6)	0.983
Pancreatitis	13 (1.9)	38 (11.4)	51 (5.2)	<0.001

PPH = postpancreatectomy hemorrhage, DGE = delayed gastric emptying. § according to the ISGPS amylase definition. *Data are presented as *n* (%), if not indicated otherwise.

## References

[1] DeOliveira M.L., Winter J.M., Schafer M., Cunningham S.C., Cameron J.L., Yeo C.J., Clavien P.A. (2006). Assessment of complications after pancreatic surgery: A novel grading system applied to 633 patients undergoing pancreaticoduodenectomy. Ann. Surg..

[2] Kamphues C., Bova R., Schricke D., Hippler-Benscheidt M., Klauschen F., Stenzinger A., Seehofer D., Glanemann M., Neuhaus P., Bahra M. (2012). Postoperative complications deteriorate long-term outcome in pancreatic cancer patients. Ann. Surg. Oncol..

[3] Hata T., Motoi F., Ishida M., Naitoh T., Katayose Y., Egawa S., Unno M. (2016). Effect of Hospital Volume on Surgical Outcomes After Pancreaticoduodenectomy: A Systematic Review and Meta-analysis. Ann. Surg..

[4] Hartwig W., Hackert T., Hinz U., Hassenpflug M., Strobel O., Buchler M.W., Werner J. (2009). Multivisceral resection for pancreatic malignancies: Risk-analysis and long-term outcome. Ann. Surg..

[5] Mussle B., Zuhlke L., Wierick A., Sturm D., Grahlert X., Distler M., Rahbari N.N., Weitz J., Welsch T. (2018). Pancreatoduodenectomy with or without prophylactic falciform ligament wrap around the gastroduodenal artery stump for prevention of pancreatectomy hemorrhage. Trials.

[6] Wellner U.F., Kulemann B., Lapshyn H., Hoeppner J., Sick O., Makowiec F., Bausch D., Hopt U.T., Keck T. (2014). Postpancreatectomy hemorrhage–incidence, treatment, and risk factors in over 1000 pancreatic resections. J. Gastrointest. Surg..

[7] Cecka F., Jon B., Subrt Z., Ferko A. (2013). Clinical and economic consequences of pancreatic fistula after elective pancreatic resection. Hepatobiliary Pancreat. Dis. Int..

[8] Winter J.M., Cameron J.L., Campbell K.A., Arnold M.A., Chang D.C., Coleman J., Hodgin M.B., Sauter P.K., Hruban R.H., Riall T.S. (2006). 1423 pancreaticoduodenectomies for pancreatic cancer: A single-institution experience. J. Gastrointest. Surg..

[9] Bassi C., Marchegiani G., Dervenis C., Sarr M., Abu Hilal M., Adham M., Allen P., Andersson R., Asbun H.J., Besselink M.G. (2017). The 2016 update of the International Study Group (ISGPS) definition and grading of postoperative pancreatic fistula: 11 Years After. Surgery.

[10] Bassi C., Butturini G., Molinari E., Mascetta G., Salvia R., Falconi M., Gumbs A., Pederzoli P. (2004). Pancreatic fistula rate after pancreatic resection. The importance of definitions. Dig. Surg..

[11] Tani M., Kawai M., Hirono S., Hatori T., Imaizumi T., Nakao A., Egawa S., Asano T., Nagakawa T., Yamaue H. (2012). Use of omentum or falciform ligament does not decrease complications after pancreaticoduodenectomy: Nationwide survey of the Japanese Society of Pancreatic Surgery. Surgery.

[12] Facy O., Chalumeau C., Poussier M., Binquet C., Rat P., Ortega-Deballon P. (2012). Diagnosis of postoperative pancreatic fistula. Br. J. Surg..

[13] Griffith D., Hanna T., Wong K., Reece-Smith A., Aroori S., Bowles M., Stell D., Briggs C. (2018). Comparison of lipase and amylase for diagnosing post-operative pancreatic fistulae. ANZ J. Surg..

[14] Fluss R., Faraggi D., Reiser B. (2005). Estimation of the Youden Index and its associated cutoff point. Biom. J..

[15] Glickman M.E., Rao S.R., Schultz M.R. (2014). False discovery rate control is a recommended alternative to Bonferroni-type adjustments in health studies. J. Clin. Epidemiol..

[16] Pulvirenti A., Marchegiani G., Pea A., Allegrini V., Esposito A., Casetti L., Landoni L., Malleo G., Salvia R., Bassi C. (2018). Clinical Implications of the 2016 International Study Group on Pancreatic Surgery Definition and Grading of Postoperative Pancreatic Fistula on 775 Consecutive Pancreatic Resections. Ann. Surg..

[17] Bassi C., Dervenis C., Butturini G., Fingerhut A., Yeo C., Izbicki J., Neoptolemos J., Sarr M., Traverso W., Buchler M. (2005). Postoperative pancreatic fistula: An international study group (ISGPF) definition. Surgery.

[18] Butturini G., Daskalaki D., Molinari E., Scopelliti F., Casarotto A., Bassi C. (2008). Pancreatic fistula: Definition and current problems. J. Hepato-Biliary-Pancreat. Surg..

[19] Banks P.A., Bollen T.L., Dervenis C., Gooszen H.G., Johnson C.D., Sarr M.G., Tsiotos G.G., Vege S.S. (2013). Classification of acute pancreatitis—2012: Revision of the Atlanta classification and definitions by international consensus. Gut.

[20] Dong X., Zhang B., Kang M.X., Chen Y., Guo Q.Q., Wu Y.L. (2011). Analysis of pancreatic fistula according to the International Study Group on Pancreatic Fistula classification scheme for 294 patients who underwent pancreaticoduodenectomy in a single center. Pancreas.

[21] Gaujoux S., Cortes A., Couvelard A., Noullet S., Clavel L., Rebours V., Levy P., Sauvanet A., Ruszniewski P., Belghiti J. (2010). Fatty pancreas and increased body mass index are risk factors of pancreatic fistula after pancreaticoduodenectomy. Surgery.

[22] Hackert T., Werner J., Buchler M.W. (2011). Postoperative pancreatic fistula. Surg. J. R. Coll. Surg. Edinb. Irel..

[23] Halkos M.E., Puskas J.D., Lattouf O.M., Kilgo P., Kerendi F., Song H.K., Guyton R.A., Thourani V.H. (2008). Elevated preoperative hemoglobin A1c level is predictive of adverse events after coronary artery bypass surgery. J. Thorac. Cardiovasc. Surg..

[24] Little S.A., Jarnagin W.R., DeMatteo R.P., Blumgart L.H., Fong Y. (2002). Diabetes is associated with increased perioperative mortality but equivalent long-term outcome after hepatic resection for colorectal cancer. J. Gastrointest. Surg..

[25] Wright C.D., Kucharczuk J.C., O’Brien S.M., Grab J.D., Allen M.S. (2009). Predictors of major morbidity and mortality after esophagectomy for esophageal cancer: A Society of Thoracic Surgeons General Thoracic Surgery Database risk adjustment model. J. Thorac. Cardiovasc. Surg..

[26] Malleo G., Mazzarella F., Malpaga A., Marchegiani G., Salvia R., Bassi C., Butturini G. (2013). Diabetes mellitus does not impact on clinically relevant pancreatic fistula after partial pancreatic resection for ductal adenocarcinoma. Surgery.

[27] Mathur A., Pitt H.A., Marine M., Saxena R., Schmidt C.M., Howard T.J., Nakeeb A., Zyromski N.J., Lillemoe K.D. (2007). Fatty pancreas: A factor in postoperative pancreatic fistula. Ann. Surg..

